# Bottom-Illuminated Photothermal Nanoscale Chemical Imaging with a Flat Silicon ATR in Air and Liquid

**DOI:** 10.1021/acs.analchem.3c04348

**Published:** 2024-03-06

**Authors:** Ufuk Yilmaz, Savda Sam, Bernhard Lendl, Georg Ramer

**Affiliations:** † Institute of Chemical Technologies and Analytics, TU Wien, Vienna 1060, Austria; ‡ Centre for Advanced Photonics and Process Analysis, Munster Technological University, Cork T12P928, Ireland

## Abstract

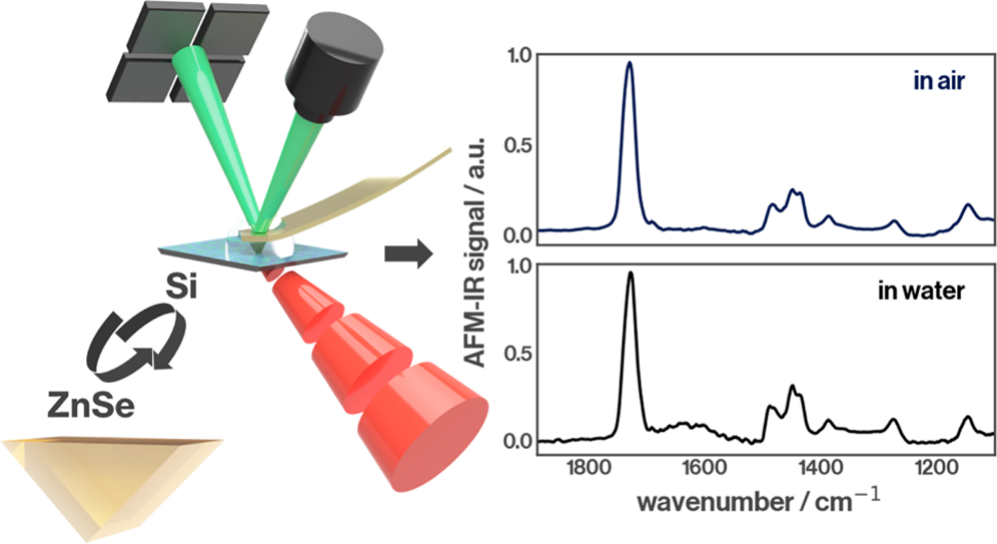

We demonstrate a novel approach for bottom-illuminated atomic force microscopy and infrared spectroscopy (AFM-IR). Bottom-illuminated AFM-IR for measurements in liquids makes use of an attenuated total reflection setup where the developing evanescent wave is responsible for photothermal excitation of the sample of interest. Conventional bottom-illuminated measurements are conducted using high-refractive-index prisms. We showcase the advancement of instrumentation through the introduction of flat silicon substrates as replacements for prisms. We illustrate the feasibility of this technique for bottom-illuminated AFM-IR in both air and liquid. We also show how modern rapid prototyping technologies enable commercial AFM-IR instrumentation to accept these new substrates. This new approach paves the way for a wide range of experiments since virtually any established protocol for Si surface functionalization can be applied to this sample carrier. Furthermore, the low unit cost enables the rapid iteration of experiments.

## Introduction

Mid-infrared (IR) spectroscopy provides information about molecular chemical composition through well-established spectra–structure correlations. Absorption of mid-IR-light stimulates vibrational modes, enabling the identification of specific functional groups. The spectral region—from 2.5 to 25 μm (4000–400 cm^–1^)—is of particular interest as it encompasses fundamental molecular vibrations associated with specific molecular features. This makes mid-IR spectroscopy a popular choice for chemical analysis. Furthermore, when used in a microscopy configuration, mid-IR spectral imaging allows one to acquire molecular chemical images. The versatility of mid-IR chemical imaging is somewhat hampered by its limited spatial resolution, which in traditional (far-field) mid-IR spectroscopy is limited by the Rayleigh criterion^1−3^ given by
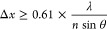
1

The long wavelength of mid-IR means that it is not usable for nanoscale measurements.

To enable optical imaging beyond the diffraction limit,^4−7^ near-field imaging techniques such as atomic force microscopy–infrared-spectroscopy (AFM-IR)^8^ or scattering–scanning near-field optical microscopy (s-SNOM)^9,10^ are employed. AFM-IR, specifically, is a hybrid technique combining atomic force microscopy and infrared spectroscopy. The working principle of AFM-IR is as follows: a pulsed, tunable IR source causes local, short-lived sample heating upon absorption of infrared light (photothermal excitation). This heating leads to local thermal expansion, which is detected using a sharp atomic force microscope probe with a ∼10–20 nm tip diameter. As first demonstrated by Dazzi et al., the transient thermal expansion of the sample results in a damped oscillatory motion of the cantilever (ring down) with amplitudes that are proportional to the sample absorption coefficient. Thus, AFM-IR provides absorbance infrared spectra that resemble conventional Fourier-transform infrared (FT-IR) spectra.^5,8^

The indirect detection scheme of AFM-IR enables not only measurements in air but also of samples in strongly absorbing media,^11,12^ such as water. This bears a huge potential for characterizing biological systems such as proteins,^13^ microorganisms, and cells in their native state. The first AFM-IR measurement obtained in water by Mayet et al.,^12^ was taking advantage of the total internal reflection of the bottom illumination configuration to reduce water absorption. Jin et al. then later demonstrated measurements on Ge prisms with poly(methyl methacrylate) samples of very thin thicknesses (∼50 nm). To achieve a reasonable SNR, the incoming beam is focused directly under a gold-coated cantilever tip.^14^ Ramer et al. demonstrated an improvement of SNR in water over measurements in air and used AFM-IR to analyze fibril secondary structure in water and heavy water.^11^

These studies all have in common that they rely on IR transparent prisms as sample carriers in bottom illumination AFM-IR geometry similar to the configuration used in the original demonstration of AFM-IR by Dazzi et al.^15^ as well as the first commercial AFM-IR systems. In this geometry, the laser beam passes through a high-refractive-index infrared transparent prism and interacts with the sample via an evanescent wave localized at the interface generated through an attenuated total internal reflection. The illumination through the prism eliminates the loss of attenuation of the pump beam in water moving toward the sample, and the attenuated total reflection concentrates the sample intensity at the surface in the sample, reducing the signal due to absorption in water. The same approach is also taken when performing s-SNOM measurements in liquid.^16^

However, bottom illumination AFM-IR (both in liquid and in air) comes with several drawbacks stemming from the size and materials of the required prisms. Only a few materials combine the required high refractive index and the infrared transparency. Typically, ZnSe (zinc selenide) and ZnS (zinc sulfide) are used, but Ge (Germanium) has also been reported. ZnSe and ZnS are not suitable for acidic samples or liquids. Furthermore, because of their shape, size, and “exotic” chemistry, these prisms are incompatible with many established surface functionalization protocols and tools. Finally, these prisms are significantly more expensive (10–100 times) than sample carriers for the more common top illumination AFM-IR illumination scheme.

In this work, we demonstrate a different approach to bottom illumination AFM-IR that is capable of measurement in water. We make use of a flat (thickness ∼0.5 mm) silicon (Si) sample carrier for bottom illumination AFM-IR in a commercial AFM-IR instrument. Through its micromachined bottom face, this type of substrate enables attenuated total reflection measurements without the need for a large prism. Through rapid prototyping, a sample holder was designed to mount the carrier. The focal length of the system was adjusted by placing a removable lens in the beam path to allow the system to be changed back to conventional sample carriers. This new system is evaluated for measurements in air and in liquid. We are confident that these sample carriers will pave the way for a broader use of AFM-IR liquid studies, not only because of the lower cost of these sample carriers compared to prisms but also because Si is a well-understood material, with good resistance against solvents and a wealth of established procedures for surface functionalization described in the literature.

## Materials and Methods

### Flat Si-ATR

**Figure 1:**
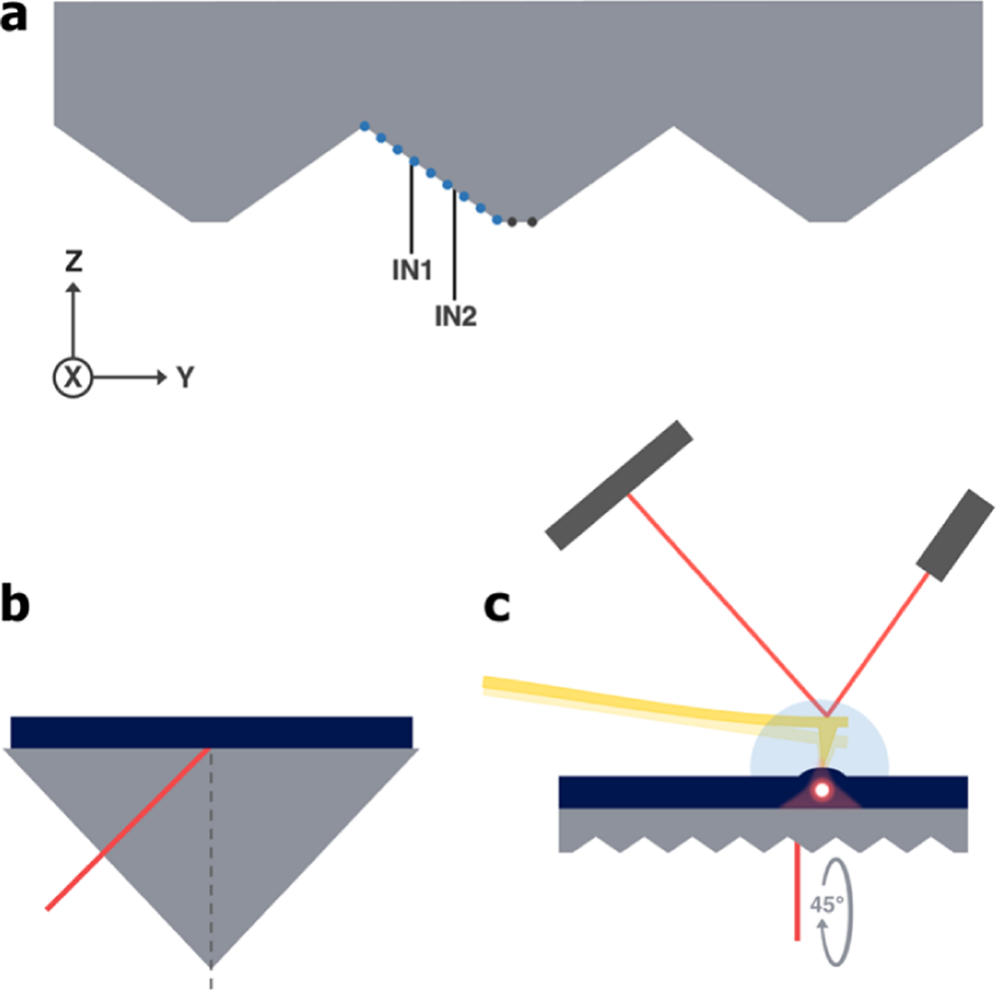
(a) Cross section of the Si-ATR with micromachined grooves. The dotted blue line visualizes the slope, while the dotted gray line represents the tip of the grooves. The slope is marked with the POI at IN1 (−197.90 μm) and IN2 (−119.78 μm). (b) Schematic representation of the AFM-IR setup utilizing the flat ATR carrier. The IR-beam is incoming at 45° from the plane of the cross section. (c) Schematic illustration of the high-refractive-index prism replaced in our work.

A flat Si-ATR (Universal Single Reflection ATR Crystal, IRUBIS GmbH, Germany) with micromachined grooves on the backside was used in this work (cross section, see Figure 1a). This Si-ATR takes advantage of grooves, which allow the coupling of IR-light, upon which there is a single bounce and subsequently an evanescent wave being formed at the surface. We will use the term “slope” to refer to the angled, machined surfaces at the bottom of the chip throughout this work. The marked points of incidence (POI) IN1 and IN2 represent the coordinates used for the simulation described later in this work. Figure 1b shows a simplified schematic of the bottom-up AFM-IR setup utilizing the new carrier. The flat ATR is replacing the high-refractive-index prism conventionally used (illustration, see Figure 1c). The IR-beam is coupled in the crystal with an outer angle of incidence of 45°, so that the plane formed by the direction incident beam and the normal of the top surface is parallel to the micromachined grooves.

### AFM-IR Instrumentation

AFM-IR data were collected on a nanoIR1 instrument (Bruker, formerly Anasys Instruments). AFM-IR experiments in air and liquid were carried out with a cantilever holder capable of performing liquid AFM measurements. An overall gold-coated contact mode cantilever (ContGB-G, BudgetSensors Innovative Solutions Bulgaria Ltd.) with a length of 450 ± 10 μm, a width of 50 ± 5 μm, and a thickness of 2 μm was used. The cantilever’s first typical resonance frequency was at 13 ± 4 kHz, with a nominal spring constant between 0.07 and 0.40 N m^–1^. The mid-IR illumination source was an MIRcat-QT, an external cavity quantum cascade laser (EC-QCL) by DRS Daylight Solutions Inc. with a spectral coverage of 1985–900 cm^–1^ (∼5–11 μm).

### Adaption of the AFM-IR System

The commercial AFM-IR system used in this work was originally optimized for ZnSe prisms. Thus, some adaptations were required to use it with flat sample carriers. This was achieved by using rapid prototyping technology (fused deposition modeling, FDM) to manufacture an adapted sample holder able to carry the flat Si-ATR (see Figure S1). The Si-ATR is held completely flat on top of the sample carrier, fixed using Kapton tape or glue. This constitutes an additional advantage of our custom-designed Si-ATR holder over conventional ATR holders which can introduce tilting of the top surface, which can affect AFM scanning. This is just due to the fact that the conventional holder relies on an open geometry while holding the prism in place with small grooves on the sides and finally using a screw to fix its position. If not done properly, there can be a tilt.

The AFM-IR system used is built for use with prisms. In this work, there is an intended change to an Si-ATR with different geometry, as well as different material; the final focusing lens in the beam path of the OEM system is not sufficient enough for high-sensitivity measurements. Taking advantage of basic optical-geometry calculations, the focal length was adjusted to the new Si-ATR geometry. This was achieved through the implementation of a removable plano-concave ZnSe lens in the focusing beam path before the final focusing lens. A kinematic magnetic mount of the plano-concave lens allowed the change between prisms and flat carriers quickly (see Figure S5 for detailed beam path).

### Sample Preparation

The polymer blend studied in this work is a mixture of poly(methyl methacrylate) (*M*_w_ = 350 K, Sigma-Aldrich) and polystyrene (*M*_w_ = 250 K, Sigma-Aldrich). As a solvent for both, toluene (AnalaR Normapur, VWR) is used. For the measurements in water, purified Milli-Q water is used. The polymer blend reference sample was created by spin coating a mixture of poly(methyl methacrylate) (PMMA) and polystyrene (PS) from solution. PMMA and PS (0.5 wt % each) were dissolved in toluene by vigorously stirring without heating. Once dissolved, the solution was directly spin-coated (Spin150i Tabletop, APF Automation) on a cleaned and dried flat silicon carrier. With 60 μL of the mixture at 1750 rpm for 60 s, an average film thickness of 250 nm was achieved. The sample was annealed for 6 h at 115 °C to allow for the local unmixing of both polymers. Film thickness was verified by using a profilometer (Bruker Dektak XT).

### IR Focus Position and Raytracing

Prior to the experiments, simulations were carried out to investigate the impact of the horizontal movement of the Si-ATR (orthogonal to the grooves, respectively, moving the stage in the *Y* direction, see Figure 3c) on the focal spot position on the surface of the Si-ATR. The objective was to determine whether such movement of the Si-ATR while scanning would result in a shift in the focal spot position as well. The beam intensity and the displacement of the focal spot on the Si-ATR surface, relative to the positions of incidence (POI, see Figure 1a), were determined by using raytracing. Raytracing was conducted using Zemax Optic Studio (ANSYS Ltd., Canada, v22.3) in nonsequential mode. The simulation model comprised the final focusing lens (*f* = 25 mm) and the Si-ATR (Figure S3). The wavelength chosen for the simulation was 6.246 μm (1601 cm^–1^). The center of the beam was determined from the maximum intensity distribution.

### Evanescent Wave Simulation

The development of the evanescent wave at the Si/air interface at the top of the carrier was simulated with finite-difference time-domain (FDTD) using an Ansys Lumerical (ANSYS Ltd. Canada, 2023 R1.1). The effects of two wavelengths relevant for the experiment, namely, 5.780 μm (1730 cm^–1^) and 6.246 μm (1601 cm^–1^), were investigated for IN1 and IN2 (see Figure 1a).

As for the used simulation parameters, the source characteristic was a Gaussian beam with 15 μm diameter directed within Si, at an angle calculated from the interface geometry and Snell’s law. The electric field is parallel to the plane of incidence. The boundary condition was set as a perfect matched layer (PML) with a standard of 32 layers. The simulation time was set to 10 ps. The selected mesh type was an auto nonuniform mesh, with an accuracy set to 3. Additionally, the mesh size was set to be overwritten near the ATR surface at a distance of 10 nm. The setup of field monitors for recording of the simulation results were placed 10 nm–1 μm from the Si-ATR surface and consisted of 10 monitors in total. These are used to monitor the field shown in Figure 7.

### AFM-IR Parameters and Data Collection

The data generated by the AFM-IR system was collected using the Analysis Studio (v3.15; Anasys Instruments) instrument control software. For the measurements, a mid-IR laser with s-polarization was used. IR maps, also called chemical imaging during this work, are maps collected at fixed wavenumber values to investigate chemical differences within the sample. Before starting an AFM-IR measurement, the mid-IR-beam position and focus plane need to be adjusted. The adjustment procedure is described in Section S1 in the Supporting Information. Measurement parameters for chemical imaging were as follows: A phase locked loop (PLL) was set to follow the resonance of the cantilever at the eigenmode around 200 kHz utilizing resonance-enhanced AFM-IR. The laser pulse width was set to 200 ns (∼4% duty cycle). The selected area for imaging was 4 μm × 4 μm with a pixel size of 600 × 300 (*xy*) and a scan rate of 0.5 lines per second (0.5 Hz). The pump laser was attenuated using a wire mesh attenuator to adjust the laser power incident on the sample for each measurement environment and each probed absorption band: In air, for the carbonyl (C=O) stretching band of PMMA (1730 cm^–1^), the pulse peak power was set to 12 mW. The peak pulse power for the C=C phenyl band of the PS (1601 cm^–1^) was adjusted to 18 mW, taking into consideration the relatively weaker intensity of this band. For IR mapping in water, the pulse peak power for the C=O stretching band was set to 190 mW, while it was set to 226 mW for the comparatively weaker C=C band.

After chemical images were taken, spectra at different sample positions were recorded to spot spectral differences of the polymer blend. Here, the cantilever probe is held at a fixed position and a spectrum of a nanoscale domain is collected by sweeping the laser wavelength (1985–900 cm^–1^). The spectra are recorded with the laser being tuned to the local contact resonance frequency of the cantilever. For the spectra, only one attenuator setting was selected for all of the wavelengths. In air, the attenuation resulted in 14 mW for 1601 cm^–1^ and 18 mW for 1730 cm^–1^ pulse peak power. In contrast, in water, it was 10 times higher due to the viscous damping. To account for the wavelength-dependent intensity of EC-QCL, spectra were normalized by an intensity spectrum collected on a power meter. AFM height images, PLL frequency, deflection images, phase images, and IR images were acquired simultaneously and in trace and retrace direction.

### Data Processing

The AFM-IR spectra and chemical images shown in this work were processed using python3 using the numpy numerical math library^17^ and matplotlib.^18^ Measurement data were exported from native NanoIR file format to a generic data format (xarray.Data set from the xarray Python package) by using the anasys-python-tools library.^19^ The spectra recorded in air and water were filtered using the Savitzky–Golay algorithm (second-order polynomial, 17 smoothing points). Spectra were normalized to a maximum value of 1. Additionally, baseline-fitting of the spectra, using the Whittaker-smoothing-based algorithm,^20^ asymmetric least-squares (ASLS), was applied. AFM-IR images were normalized by scaling the image intensity values to the range of [0,1], but they were not otherwise processed or smoothed.

## Results and Discussion

Since the predominant part of the work on bottom-illuminated AFM-IR has been carried out with ZnSe prisms under a 45° angle of incidence, we investigate the properties of the Si-ATR in comparison to those of the conventional approach. In this section, we compare conventional and Si-ATR using optical calculations to determine depth of penetration and FDTD to model the evanescent field. Furthermore, we use raytracing to understand the effect of the Si-ATR’s grooves on the focus position and demonstrate its viability for bottom-illuminated AFM-IR measurements experimentally.

### Depth of Penetration

Changing to a substrate of different refractive index also induces a change in depth of penetration (*d*_p_) of the developed evanescent wave on the surface according to (eq 2),^21^ where λ is the wavelength of the illumination source, *n*_1_ is the refractive index of the ATR, *n*_2_ is the refractive index of the sample medium, and θ is the angle of incidence.
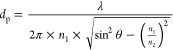
2

**Figure 2:**
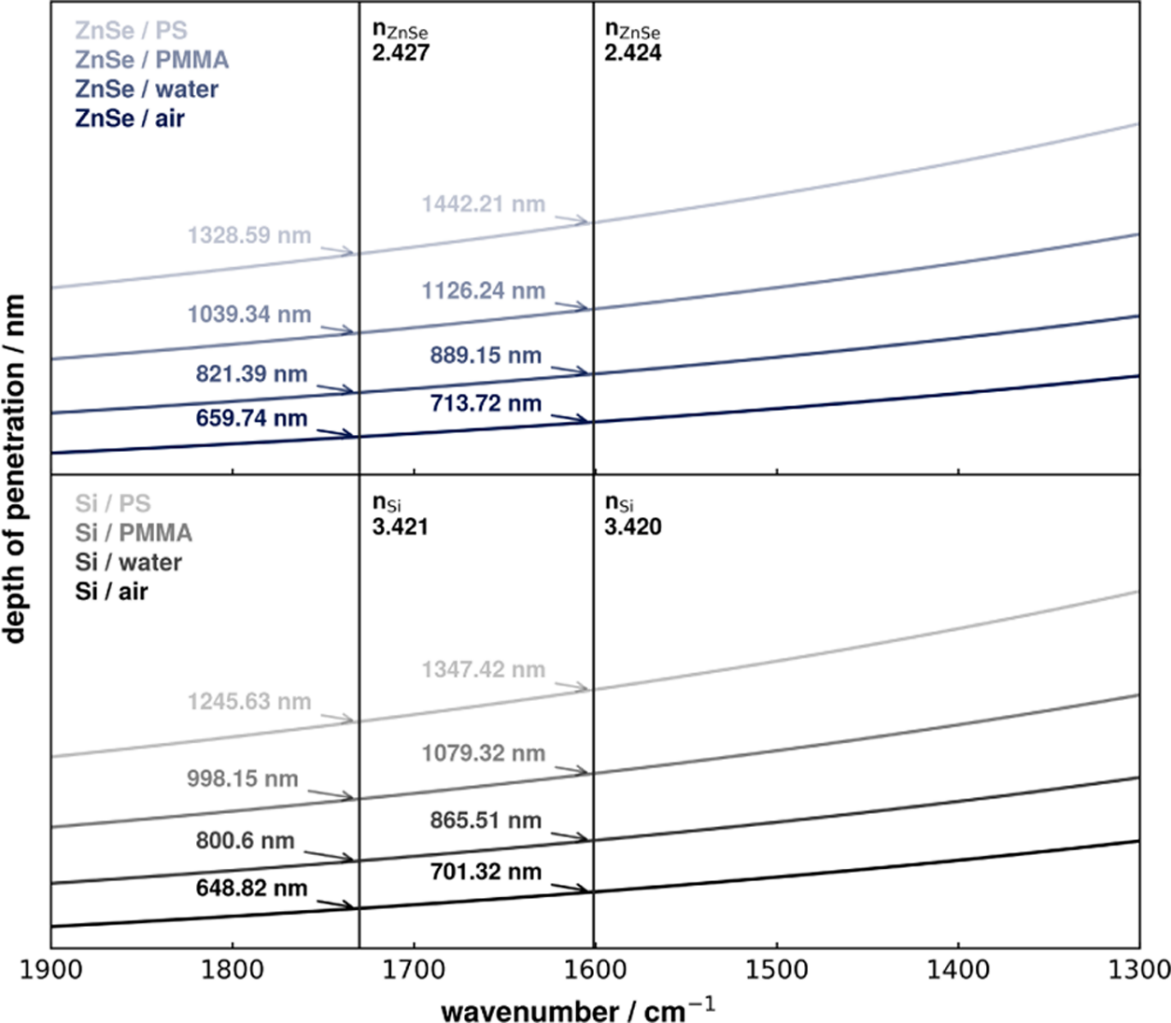
Depth of penetration (*d*_p_) of the evanescent wave in relation to the wavenumber, comparing the ZnSe prisms and flat Si-ATR in the AFM-IR bottom illumination geometry, in both air and water. The change of refractive index with wavelength is considered, but absorption in the sample layer is ignored. For ZnSe prisms, an angle of incidence of 45° is used. For the flat Si-ATR, the angle of incidence is set to 30.48°, considering the refraction of the free space beam at grooved side of the Si-ATR.

While the large difference in refractive indices (*n*_ZnSe_ ≈ 2.4 and *n*_Si_ ≈ 3.4) would suggest a much smaller depth of penetration for Si, this is not observed in the flat Si-ATR in the AFM-IR illumination geometry. This can be explained because the refraction at the side of the grooves leads to an (inner) angle of incidence of 30.48° for the Si while the angle of incidence for ZnSe is 45°. The refractive index data used for the calculation was taken from the literature.^22,23^ The theoretical depth of penetration into air, water, PMMA, and PS was calculated for both substrates, and similar *d*_p_ values were reached for both substrates (see Figure 2). This gives a theoretical estimation of the *d*_p_ in sample layers and suggests that the limit of the sample layer thickness is about 1 μm. The contribution of water to the overall absorption in dependence of the layer thickness for both conventional ZnSe-ATR and Si-ATR can be seen in the Supporting Information (see Figure S2).

### IR Focus Position and Raytracing

The AFM-IR signal amplitude is proportional to the local pump beam intensity. Hence, the relative position of the pump laser focus and the AFM tip apex and the pump beam focal spot size need to be kept constant during a measurement. This is the reason why AFM-IR instruments are typically sample scanning instruments. The conventional prism-based bottom illumination AFM-IR design is engineered to minimize the effect of scanning the prism on the focus position and spot size: by using a normal incidence of the beam onto the angled side of the sample carrier, the beam is not refracted, ensuring that it will go through the tip position even when moving the prism. When the prism is moved along the plane of incidence of the beam, the optical path length inside the high-refractive-index material changes, leading to a change in the focal plane. This is usually compensated for in large range movements by moving the focusing lens along the beam path, so the focal plane remains at the surface of the substrate. In the machined Si-ATR, no compensation of focal plane is needed while scanning, as the optical path length (<0.5 mm) inside Si is negligible compared to the focal length of the focusing lens (>20 mm). In contrast to the conventional prism sample carrier, normal incidence of the probe beam is not possible in the Si-ATR. The beam is always incident at an angle other than 90° onto the faces of the machined grooves and the direction of propagation is thus changed at the air–Si interface. This means that scanning the prism laterally (along *Y* in Figure 1a), orthogonal to the direction of the grooves, will lead to a shift of the focus position.

**Figure 3:**
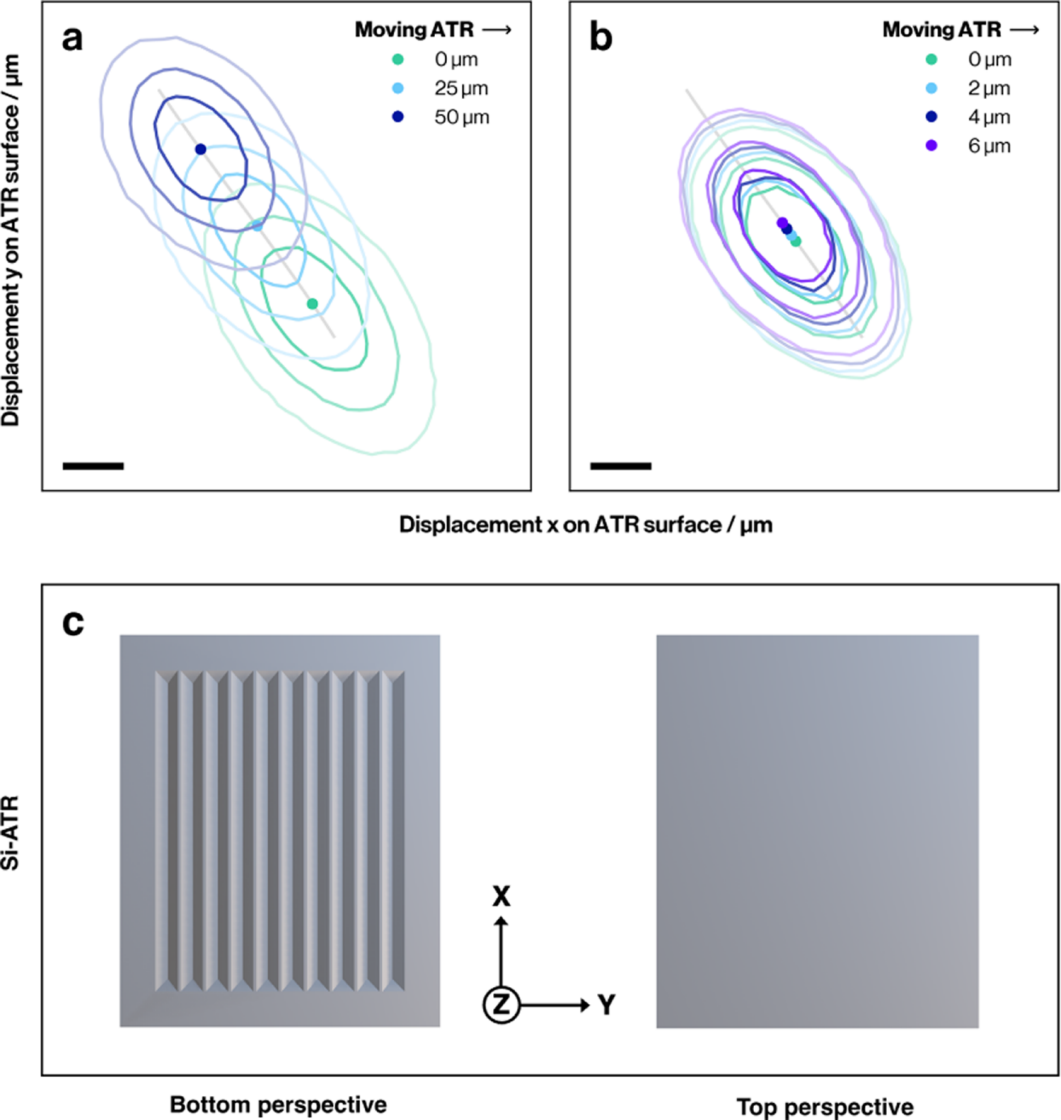
Beam intensity distribution (simulated) and positional displacement of the focal spot on the surface of the Si-ATR when moving the chip orthogonal to the grooves (in *Y* direction) at a wavelength of 1601 cm^–1^ for the case of (a) large scale movement of 25 μm steps, as needed for selecting a sample position (b) and smaller movements of 2 μm typical within an AFM-IR image. The raytracing is performed at the slope between IN1 and IN2 (see the gray line in panels (a, b)). This is described before (see Figure 1a). (c) Illustrative bottom and top perspective of Si-ATR. Scale bars are 5 μm.

Using nonsequential raytracing to model the intensity distribution at the surface of the Si-ATR, we see that, indeed, when the sample is moved sideways in a direction orthogonal (in *Y* direction, see Figure 3c) to the grooves, by a total of 50 μm, the focal spot moves by <8 μm for each step (25 μm) relative to the original position (see Figure 3a). However, when moving sideways by distances typically used for AFM imaging (see Figure 3b), the shift for each step is much smaller (<0.7 μm). In this more realistic scenario for AFM-IR imaging, we see negligible intensity changes at the surface, which would be removed by typical AFM-IR postprocessing such as spectral normalization or calculation of band ratios. However, when moving larger distances (e.g., when switching between different sample locations), the beam pointing potentially needs to be readjusted.

### Evanescent Wave

**Figure 4:**
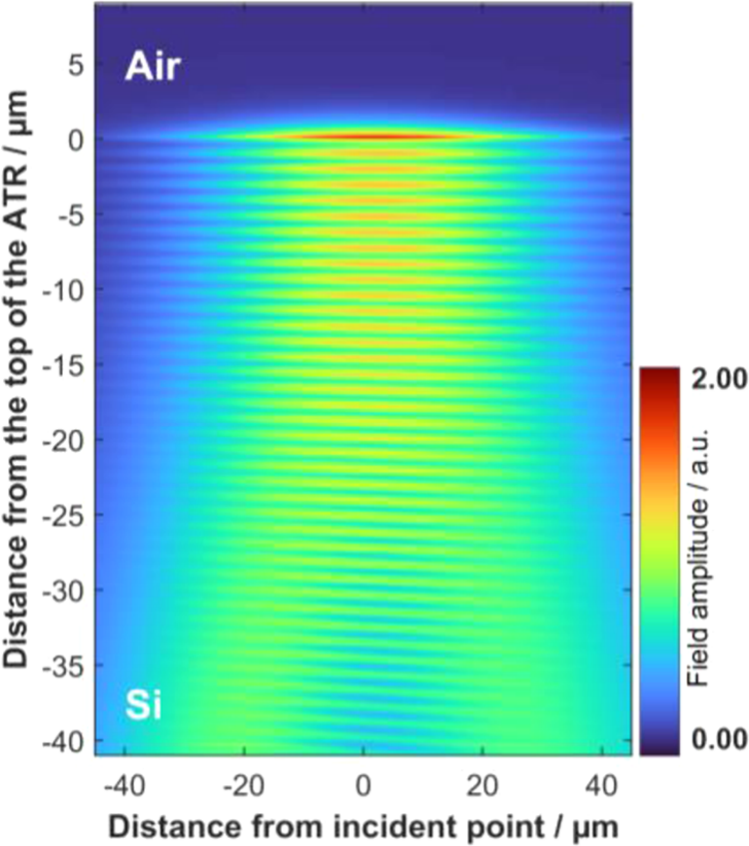
Normalized field amplitude for IN1 at 1601 cm^–1^ (6.246 μm). After reflection, an evanescent wave is developed at the crystal surface. The depth of penetration seen is *d*_p_ = 713 nm.

Using FDTD, we calculate the propagation of light in the Si-ATR. After reflection at the top surface, an evanescent wave develops (see Figure 4) and interference of the incident and reflected waves shows the expected pattern of a standing wave. Varying the point of incidence and the wavelength in the simulation allows us to study the effects of these parameters on the surface field intensity and the depth of penetration. (All field intensities in this section are normalized to unit incident intensity).

**Figure 5:**
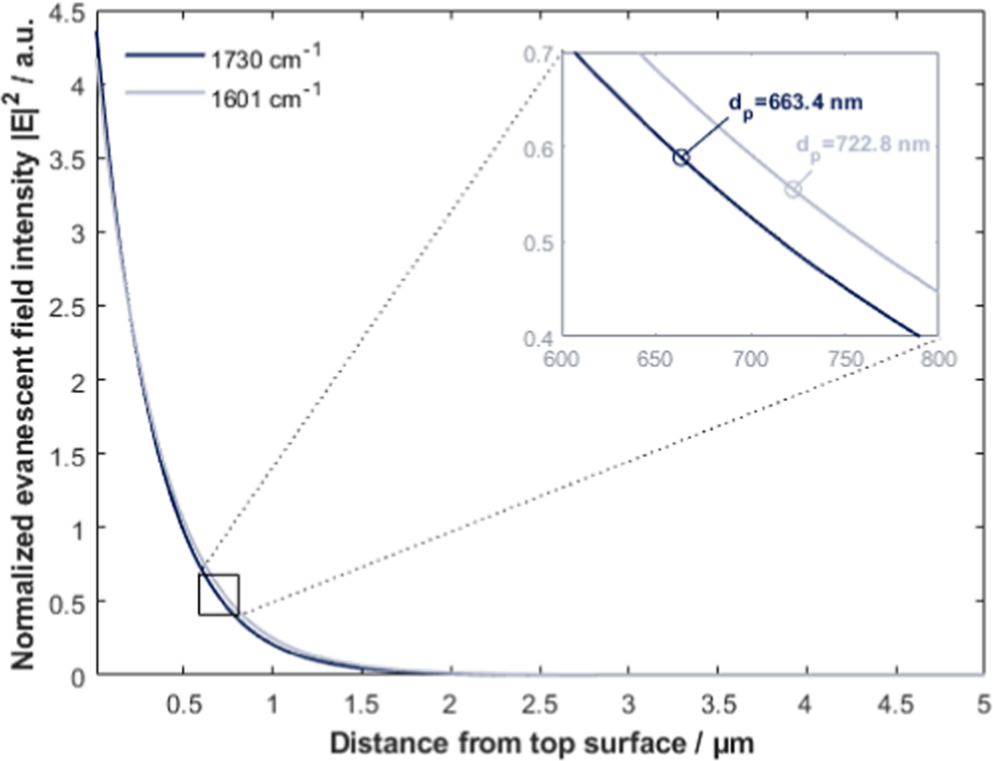
Evanescent field amplitude at IN1. The inset is an enlargement of the graph at 1/*e* and shows the depth of penetration for 1730 cm^–1^ (*d*_p_ = 722.8 nm) and 1601 cm^–1^ (*d*_p_ = 663.4 nm). Note that for better visibility, the *x*-axis of the inset is converted from μm in nm.

When the wavelength is varied, the expected effect on the depth of penetration is observed (i.e., shorter wavelength leading to lower depth of penetration as in Figure 5). The depth of penetration of the evanescent wave developed at two simulated wavenumbers is *d*_p_ = 722.8 nm for 1730 cm^–1^ and *d*_p_ = 663.4 nm for 1601 cm^–1^. This is in good agreement with our calculations using (eq 2). The wavenumber has a minimal effect on the intensity of the evanescent field at the surface (see Figure 5). The normalized field intensity at the surface observed is ∼0.59 for 1730 cm^–1^ and ∼0.56 for 1601 cm^–1^. This slight difference can be explained by a higher absorption in Si at the lower wavenumber.

The evanescent field intensity at the surface is close to four times higher than the incoming field intensity. This intensity enhancement effect in total internal reflection close to the critical angle is well-known (see the review of Martin-Fernandez et al.^24^ for a theoretical discussion).

**Figure 6:**
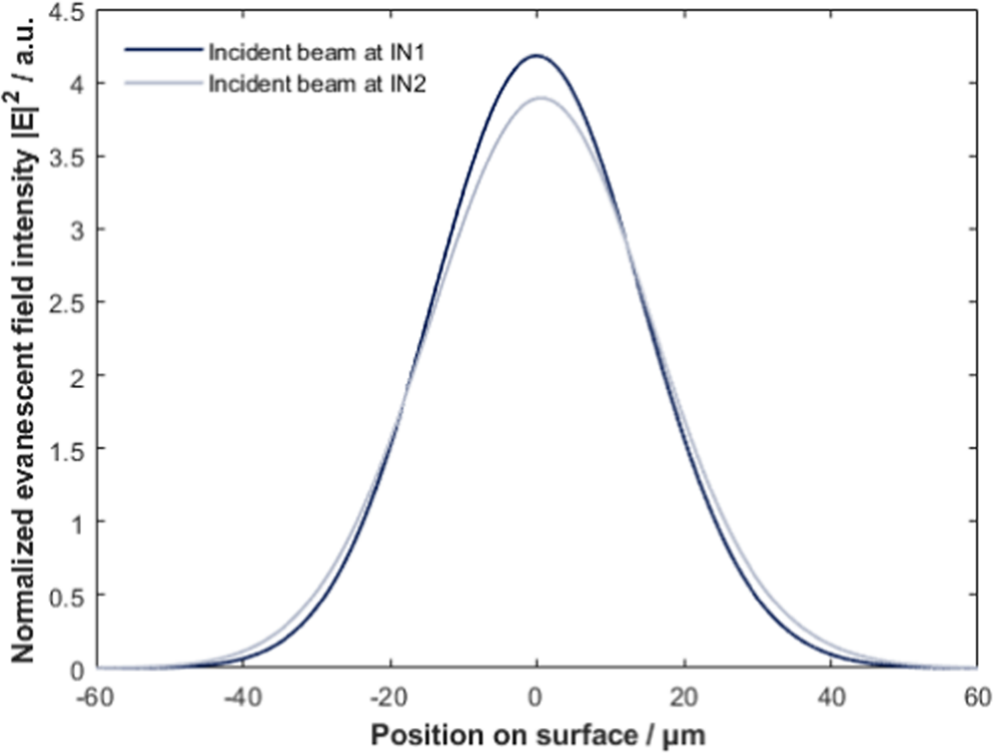
Intensity difference of evanescence wave generated by incident beam at points IN1 and IN2. The difference is mainly due to different propagation loss of different path length. Calculated for excitation: 1601 cm^–1^. The lateral intensity distribution of the evanescent field does not change with distance from the surface (see Figure 7).

Our FDTD simulations show changes in the normalized maximum intensity of the evanescent field for different points of incidence (see Figure 6). For incidence, close to the center of the groove IN1, the intensity is higher 4.18 compared to 3.89 at IN2 which is further from the groove. This can be explained by different propagation losses at different pathlengths in Si.

It should be noted that no evanescent field can be generated when the beam is incident at the tip of a groove, as this part of the chip is parallel to the top surface. The bottom of the groove also needs to be avoided for practical reasons: here, the beam is spatially split, leading to two focal spots far apart. However, when these areas are avoided, the results of the FDTD study confirm that for real-world sample preparation and measurement situations, the carriers should perform optically similar to conventional ZnSe prisms.

### AFM-IR Imaging and Spectroscopy on Si-ATR in Air

The AFM-IR measurements in air and water were collected from a 4 μm × 4 μm area on the Si-ATR, with the polymer blend, as described previously.

**Figure 7:**
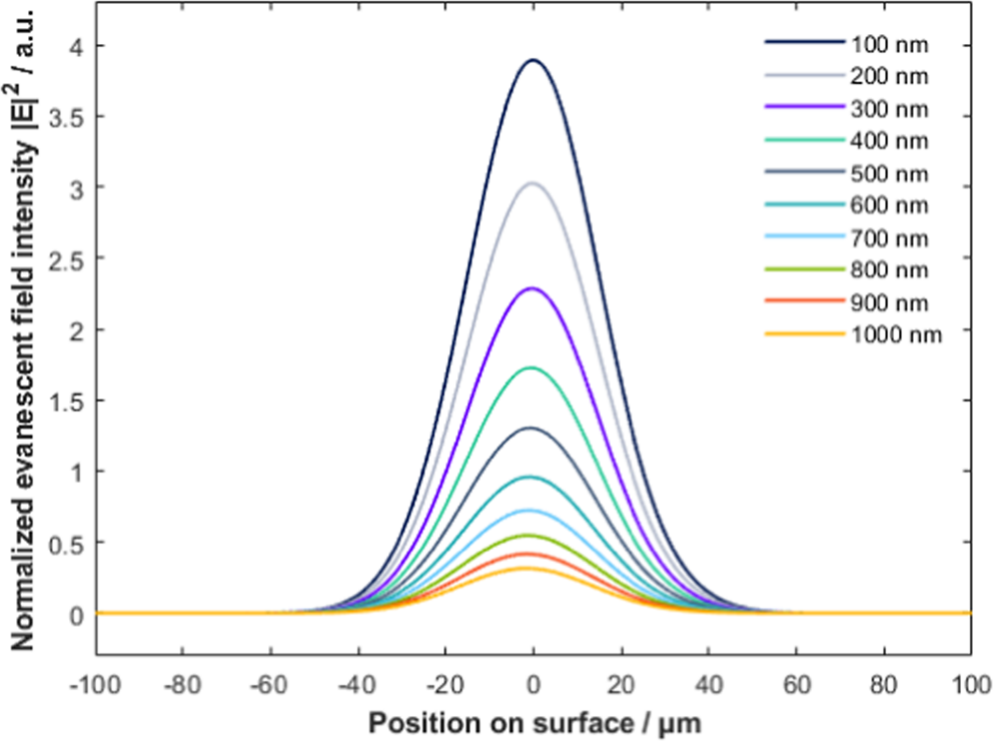
Evanescent field intensities at different heights above the ATR crystal top surface at incident point IN1 at 1601 cm^–1^ (6.246 μm). From 100 to 1000 nm with 100 nm step increments (from tallest to lowest peak).

**Figure 8:**
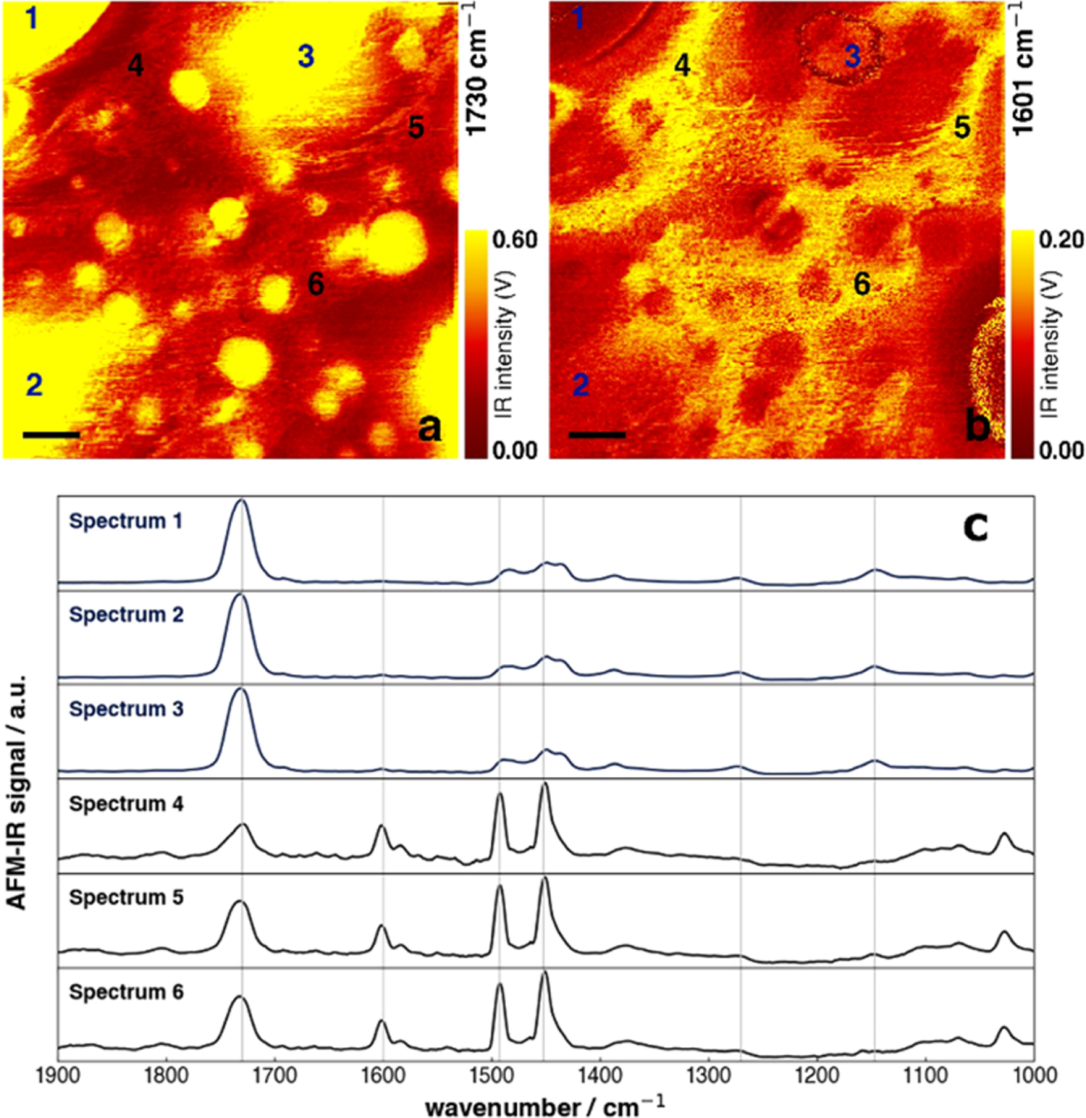
IR images of the same 4 μm × 4 μm sample area, recorded in air at (a) 1730 cm^–1^ (C=O stretch) and (b) 1600 cm^–1^ (C–C ring-stretch), respectively, exhibiting PMMA-rich and PS-rich phases. The indices mark locations where spectra were recorded and plotted in panel (c). Scale bars are 500 nm.

The wavenumbers selected for the chemical imaging are 1730 cm^–1^ for PMMA, corresponding to the carbonyl stretching (C=O) band and 1601 cm^–1^ corresponding to the C=C phenyl stretching of PS. Therefore, in Figure 8a, areas of higher intensity (yellow) are PMMA-richer, whereas the areas with less intensity are PS-richer regions. This is also double checked with the IR image recorded at 1601 cm^–1^ (see Figure 8b). Furthermore, this is verified by infrared spectra recorded at different locations. Within the sample, phase separation can be observed, although the blend is not a constituent of distinct phases but a mixture of PMMA and PS.

The infrared spectra in Figure 8c furthermore underline the PMMA-rich (see spectra 1, 3, and 5) and PS-rich (see spectra 2, 4, and 6) phases. Generally, a predominance of PMMA is observable, whereas the intensity of PS is lower due to the weak nature of the aromatic ring breathing vibration (1601 cm^–1^). The vertical lines correspond to the specific absorption bands of the PMMA and PS: at 1730 cm^–1^ for PMMA and 1601 cm^–1^, 1494, and 1452 cm^–1^ for PS. The corresponding AFM topography image can be found in the SI (see Figure S4).

### AFM-IR Imaging and Spectroscopy on Si-ATR in Water

**Figure 9:**
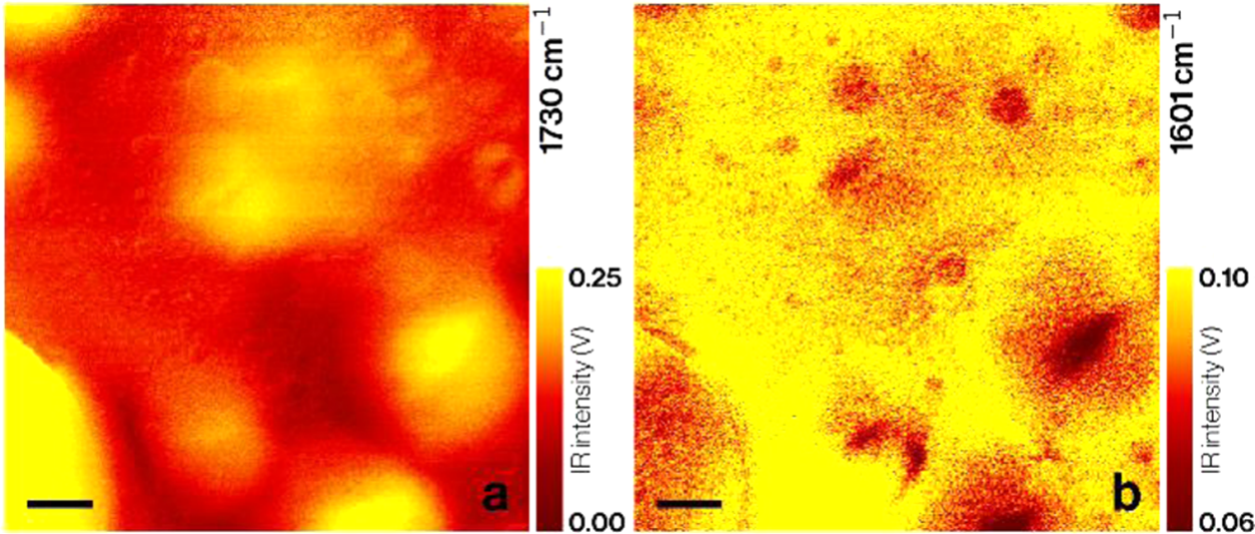
IR images of the same 4 μm × 4 μm sample area, recorded in water at (a) 1730 cm^–1^ (C=O stretch) and (b) 1600 cm^–1^ (C–C ring-stretch) respectively. Scale bars are 500 nm.

IR images in water are depicted in Figure 9, where (a) is recorded at 1730 cm^–1^ and (b) at 1601 cm^–1^. The signal amplitude in these measurements is lower than in air due to the damping of the mechanical oscillation in water.^11^ Nevertheless, again, here, two polymer phases can be distinguished.

**Figure 10:**
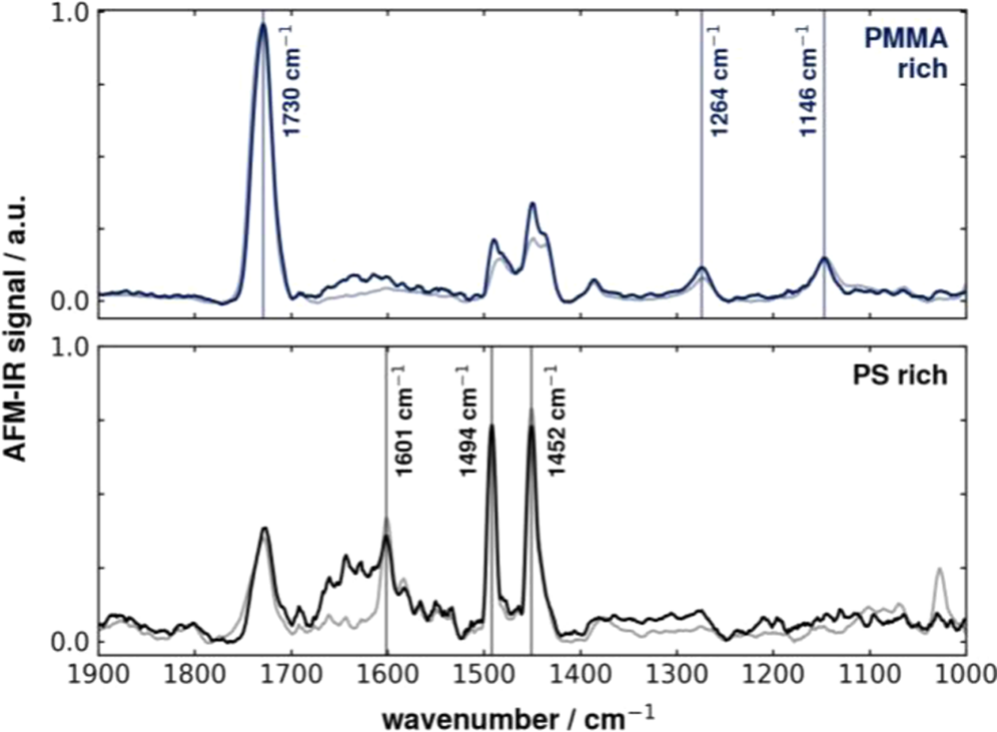
Nanoscale infrared spectra recorded in PMMA-rich (top) and PS-rich (bottom) areas. Each both, in air and water, where the lighter line represents the spectrum measured in air and the darker line represents measurement in water. Spectra are normalized and baseline-corrected.

Spectra for PMMA and PS in both air and water are depicted in Figure 10. The spectra gathered in a PMMA-rich phase of the sample are presented in Figure 10 (top) and normalized for comparability. The most intense peak is a characteristic band of PMMA and is assigned to the carbonyl (C=O) stretching vibration. At 1146 and 1264 cm^–1^, the C–O stretch forms additionally and is therefore a further indicator for PMMA. However, as previously discussed, the sample polymer blend does not consist of two distinct single phases rather than a mixture of both phases. For this reason, in the spectra, we can see features of PS like the weak band of the aromatic ring stretching at 1601 cm^–1^. There is a good match between the spectra measured in air (dark blue line) and those measured in water (light blue line). Here, additional features are visible in the measurements in water, namely, the broad band between about 1520 and 1680 cm^–1^. This is due to the absorption of water itself and caused by the evanescent wave reaching in the water above the sample surface and causing photothermal expansion and therefore being detected by the cantilever.

Also, in Figure 10 (bottom), spectra for a PS-rich region are recorded and normalized for comparability. Here, again the darker line corresponds to the spectra recorded in air, whereas the lighter line corresponds to spectra recorded in water. For imaging PS, the aromatic ring breathing modes are used. These appear at 1601, 1494, and 1452 cm^–1^, respectively. For the marker bands of the polystyrene, we can observe a good match between the measurements in air and water, for both band position as well as normalized signal intensity. The broad absorption band due to water can also be observed in the PS-rich region. Nevertheless, the 1601 cm^–1^ peak stands out.

## Conclusions and Outlook

In this work, a new type of ATR sample carrier for AFM-IR experiments was introduced and successfully tested. The sample carrier is compatible with a common commercial AFM-IR instrument, paving the way for cost-effective nanoscale chemical imaging in air and in liquids.

Using raytracing and photonic simulations, we verified that the flat, Si-ATR carrier is suitable for AFM-IR measurements and should provide results similar to conventional ZnSe prism sample carriers.

Experimentally, we have demonstrated the feasibility of our approach by using a simple polymer blend consisting of PMMA and PS. We have also shown that chemical imaging and mid-IR spectroscopy are possible at the nanoscale with a flat, Si-ATR.

Going forward, we believe that this novel approach allows conducting AFM-IR measurements in liquids that would not have been possible otherwise, i.e., of samples in or on acidic environments. Furthermore, silicon surface functionalization is well understood and opening the door for further studies of thin films, selective enrichment of analytes on the substrate, or affinity-capture of biomolecules, vesicles, or microorganisms.

To use the Si-ATR for more complex applications then showcased in this work, some practical limitations will have to be addressed. When focusing the IR-beam, it is crucial to avoid the tips of the grooves because they have a small flat interface that is a parallel surface and thus prevent the generation of an evanescent field. Additionally, the bottom of the groove should be avoided to prevent beam splitting and the subsequent spatial separation of focal spots. The range between tip and bottom is >300 μm, leaving a large scan range to be used. Furthermore, improved mounting of the Si-ATR using a low-thermal expansion metal holder and a mechanism for reproducible positioning of the chip should be devised for more stable measurements.

## Data Availability

Raw data, data processing and data visualization for Figures 2, 3, and 8–10 including the required python environment are available on Zenodo in the form of a Docker container (https://zenodo.org/doi/10.5281/zenodo.8366243).

## Supporting Information Available

Additional figures S1–S5 and a description of the initial optical alignment of the system (PDF)
