# Supplementary material for: Bottom-Illuminated Photothermal Nanoscale Chemical Imaging with a Flat Silicon ATR in Air and Liquid
Source: Anal Chem. 2024 Mar 6;96(11):4410–8. doi: 10.1021/acs.analchem.3c04348 (PMC10955511; doi:10.1021/acs.analchem.3c04348)
Supplement: Supplementary file 1 — ac3c04348_si_001.pdf [file ac3c04348_si_001.pdf]

Supporting Information for

Bottom-illuminated photothermal nanoscale chemical imaging with a flat silicon ATR in air and liquid

Ufuk Yilmaz, [†] Savda Sam, [†‡] Bernhard Lendl, [†] and Georg Ramer [†*]

† Institute of Chemical Technologies and Analytics, TU Wien, Vienna, Austria.

‡ Centre for Advanced Photonics and Process Analysis, Munster Technological University, Cork, T12P928, Ireland

1. ALIGNMENT OF MID-IR BEAM TO THE AFM TIP POSITION

The experiment requires an initial alignment of the IR laser to the position of the AFM tip. This was carried out in four steps. Several actuators of the nanoIR1 system are involved in this process. These are:

1. Sample positioner, a stepper motor-controlled stage that moves the sample holder in three dimensions.
2. Scan stage: a piezoelectric scanner moving the sample laterally and tip vertically for imaging.
3. Beam positioning and focusing unit, consisting of two manual micrometer screws that shift folding mirrors inside to move the beam path laterally, another micrometer that moves the final focusing lens of the instrument to adjust the focus plane, and a stepper motor that also drives the micrometer for fine adjustment of the focus.
4. Beam steering mirrors: two tilting mirrors that adjust the angle of the beam for fine adjustment of beam pointing.

Rough alignment of mid-IR beam

By using a visible guide beam co-axial with the IR beam, the beam position was roughly adjusted to the position of tip. As the Si carrier is intransparent to visible light the sample positioner was used to move the edge of carrier to be positioned under the AFM tip. Then manual micrometer screws of the beam positioning unit were used to shift the beam along the carrier’s edge until the guide beam was scattered at edge of carrier.

Selection of driving frequency for resonant excitation

The sample carrier was then used again to move the sample, so its center was roughly under the tip. The AFM tip was approached to the surface and contact mode AFM imaging was used to select a part of the sample with an absorber. Then, the tip was help on that absorber and a thermal noise spectrum of the tip was collected (called “Thermal Tune” in the Analysis Studio software). The exact resonance frequency of one of the contact resonances of the tip on this surface was determined from this thermal noise spectrum.

Coarse alignment of the mid-IR beam

The repetition rate of the mid-IR source was set to the contact resonance frequency determined in the previous step and its wavelength was set to one of the absorption bands of the sample. The beam path was shifted laterally using the beam positioning unit until the AFM-IR signal had reached a maximum. Then the focus plane was adjusted using the beam focusing micrometer screw, again towards the maximum. Adjustment of lateral position and focus plane were repeated several times until no further increase in AFM-IR signal amplitude could be achieved. During this process, in some cases, it was necessary to increase the attenuation of the mid-IR beam and sometimes check for drift of the sample by scanning the AFM topography image again.

Fine alignment of the mid-IR beam and compensation of beam pointing

In the final step of the alignment, the beam steering mirrors were used to scan the mid-IR beam in a 2D grid and the AFM-IR signal was recorded while scanning. The mirror positions for maximum AFM-IR signal were stored. This step provides a finer adjustment of beam positioning than the manual shifting of the beam and compensates for differences in pointing between different chips of the EC-QCL source. Hence, mirror positions need to be stored for each of the four chips of the source.

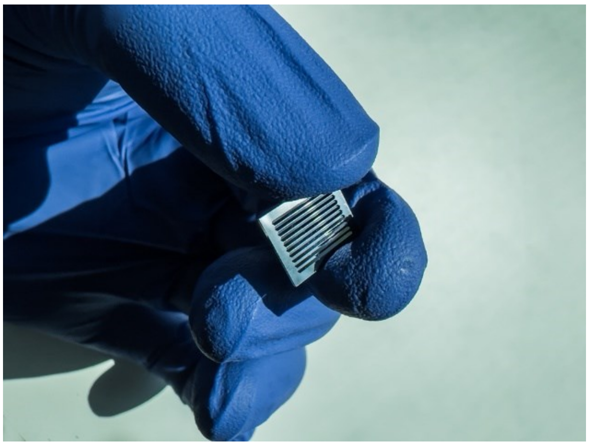

a)

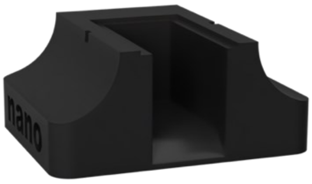

b)

Figure S1 a) Photo of the Si-ATR (grooves) and b) 3D-Model of the Si-ATR holder

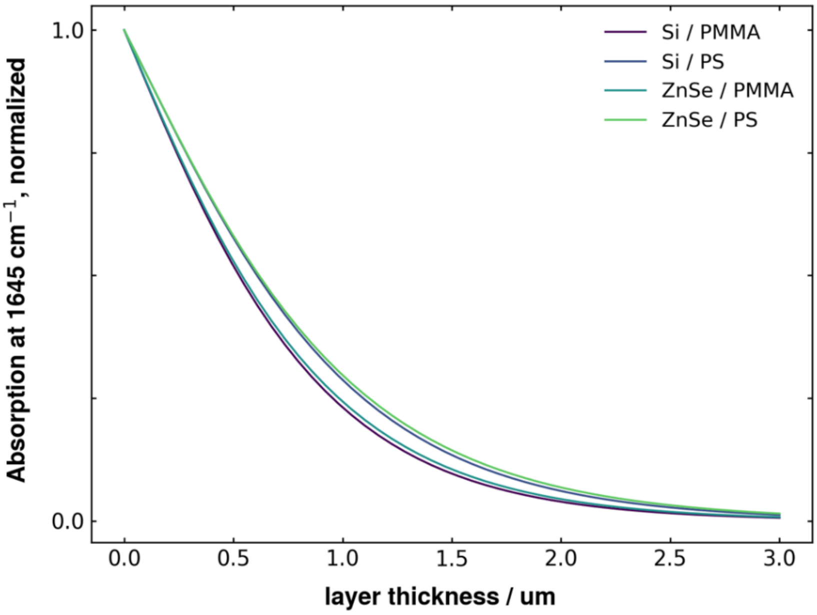

Figure S2 We calculate the contribution of water absorption to the overall absorption in the spectrum in dependence of layer thickness for conventional and Si-ATR for two different polymers. To better show the behavior, all curves are normalized to the absorption measured at zero depth. The imaginary parts of the refractive index for PMMA and PS were set to 0, hence all absorption in the curves is due to the water deformation band. As can been seen the curves for ZnSe and Si prism are almost identical. It also needs to be noted that the contribution of water absorption by water will likely be lower than seen here, if there is no direct excitation of the cantilever and the signal only stems from the sample below the tip.

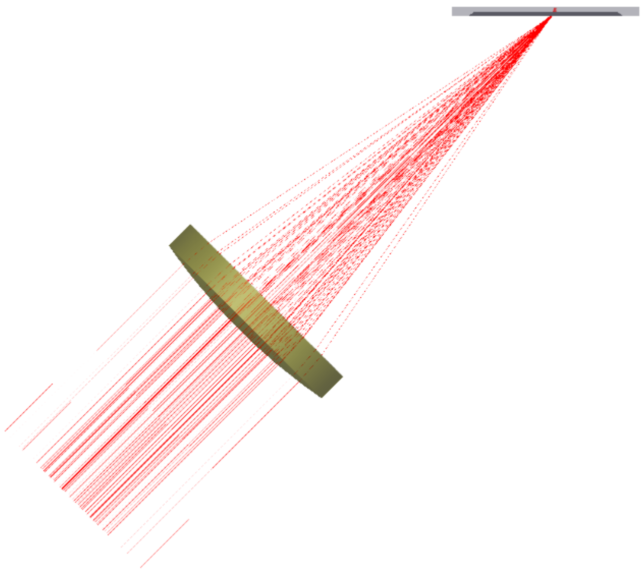

Figure S3 Zemax Raytracing simulation illustration of the Si-ATR with IR illumination in 45-degree angle trough an 1/2” focusing lens

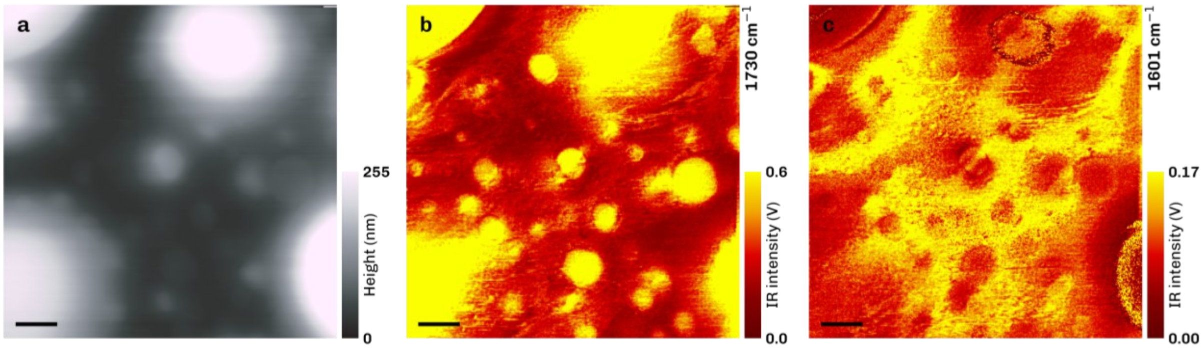

Figure S4 AFM-IR measurements: Images of the same 4 x 4 µm sample area, b) recorded in air at a) 1730 cm^{-1} (C=O stretch) and c) 1600 cm^{-1} (C-C ring-stretch) respectively, exhibiting PMMA rich- and PS rich-phases. The indices mark locations where spectra were recorded and plotted in c). Scale bars are 500 nm.

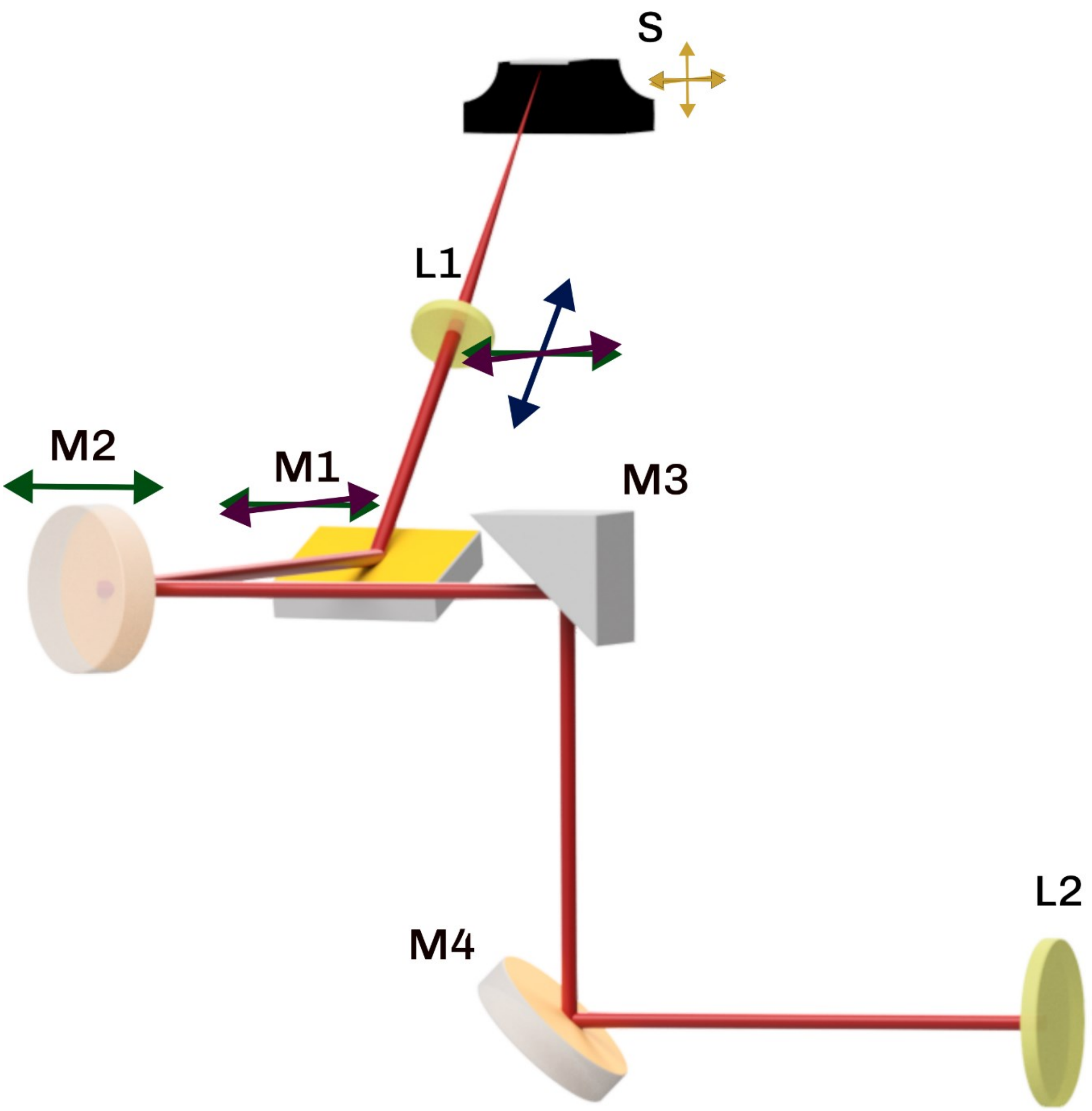

Figure S5 Rendering of the beam path in the modified bottom illumination AFM-IR setup. The IR beam first passes concave lens L2, then is folded by gold mirrors M4 and M3 onto the first moving mirror M2. This mirror moves along the beam path (along the green arrow next to M2) and shifts the beam laterally. The second moving mirror M1 moves laterally together with M2 (green arrow under M1) and at a right angle to the direction of movement of M2 (red arrow under M1). At M1 the beam is reflected upwards at an angle of 45 ° and passes the focusing lens L1. This lens moves laterally together with M1 and M2 (green and red arrows) and, independently of them along the beam path (blue arrow next to L1) to change the focal plane. In the initial setup of a measurement the M1, M2 and L1 are shifted for coarse alignment of the focal position to the position of the tip. The sample holder S moves independently from M1, M2 and L1. A stepper stage moves it vertically and laterally (yellow arrows). For AFM imaging, the sample is moved laterally by a piezo actuator. The vertical motion during AFM imaging is carried out by the AFM tip, rather than the sample. L2 can be removed to switch back to measurements on ZnSe prisms.
